# Regioselective C4 and C6 Double Oxidation of Cellulose by Lytic Polysaccharide Monooxygenases

**DOI:** 10.1002/cssc.202102203

**Published:** 2021-12-18

**Authors:** Peicheng Sun, Christophe V. F. P. Laurent, Vincent J. P. Boerkamp, Gijs van Erven, Roland Ludwig, Willem J. H. van Berkel, Mirjam A. Kabel

**Affiliations:** ^1^ Laboratory of Food Chemistry Wageningen University & Research Bornse Weilanden 9 6708 WG Wageningen (The Netherlands; ^2^ Biocatalysis and Biosensing Laboratory Department of Food Science and Technology BOKU-University of Natural Resources and Life Sciences Muthgasse 18 1190 Vienna Austria; ^3^ Institute of Molecular Modeling and Simulation Department of Material Sciences and Process Engineering BOKU-University of Natural Resources and Life Sciences Muthgasse 18 1190 Vienna Austria

**Keywords:** isotopic labeling, lytic polysaccharide monooxygenase, mass spectrometry, oligosaccharides, regioselectivity

## Abstract

Lytic polysaccharide monooxygenases (LPMOs) play a key role in enzymatic degradation of hard‐to‐convert polysaccharides, such as chitin and cellulose. It is widely accepted that LPMOs catalyze a single regioselective oxidation of the C1 or C4 carbon of a glycosidic linkage, after which the destabilized linkage breaks. Here, a series of novel C4/C6 double oxidized cello‐oligosaccharides was discovered. Products were characterized, aided by sodium borodeuteride reduction and hydrophilic interaction chromatography coupled to mass spectrometric analysis. The C4/C6 double oxidized products were generated by C4 and C1/C4 oxidizing LPMOs, but not by C1 oxidizing ones. By performing incubation and reduction in H_2_^18^O, it was confirmed that the C6 *gem*‐diol structure resulted from oxygenation, although oxidation to a C6 aldehyde, followed by hydration to the C6 *gem*‐diol, could not be excluded. These findings can be extended to how the reactive LPMO‐cosubstrate complex is positioned towards the substrate.

## Graphical abstract

**Single and double**? Lytic polysaccharide monooxygenases (LPMOs) were reported to form C1/C4 double oxidized cello‐oligosaccharides in addition to C1 or C4 single oxidized ones. Using NaBD_4_ reduction and HILIC‐ESI‐CID‐MS/MS^2^ approach, it is found that C4/C6 double oxidized cello‐oligosaccharides are generated, and not C1/C4 double oxidized products. Experiments in H_2_^18^O further show that C6 *gem*‐diol is formed by an LPMO‐catalyzed reaction.

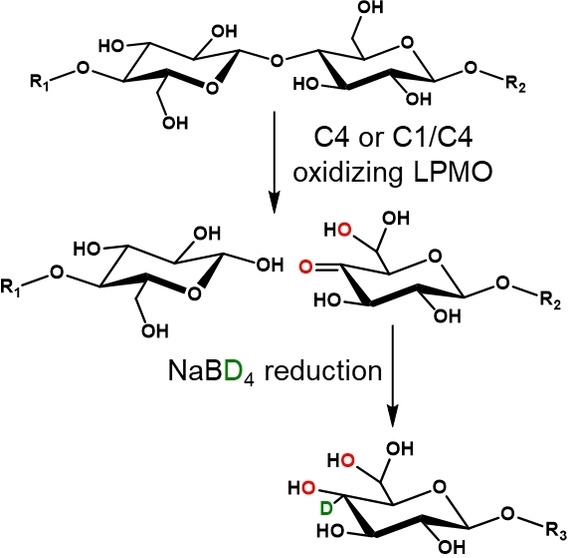

## Introduction

In context of fungal physiology, biocatalysis, and biorefinery processes, lytic polysaccharide monooxygenases (LPMOs) are beneficial to the enzymatic degradation of hard‐to‐convert polysaccharides, such as cellulose and chitin, and, simultaneously, introduce oxidized functional groups.^[1]^

LPMOs are classified as auxiliary activities (AA) families in the Carbohydrate‐Active enZyme (CAZy) database.^[2]^ Based on amino acid sequence similarity, LPMOs are currently categorized in AA9–AA11 and AA13–AA17.^[3]^ So far, AA9 is the largest LPMO family, in which all candidates are from fungal origin, and all characterized LPMOs are oxidatively cleaving cellulose.^[4]^

LPMOs exploit a mono‐copper‐dependent active site, where the copper is coordinated by a unique histidine brace.[^1d^, ^5^] Although part of the LPMO's mono(per)oxygenase mechanisms remain under debate, it is generally accepted that an external reductant and a cosubstrate (O_2_ or H_2_O_2_) is required for catalysis.[^4^, ^6^] LPMOs catalyze a regioselective single oxidation reaction, of either the C1 or the C4 position in the glycosidic linkage, which results in cleavage of this destabilized linkage.^[4a]^ In addition to non‐oxidized products, C1 oxidation generates δ‐lactones, while after C4 oxidation 4‐ketoaldoses are formed. In aqueous solutions, however, δ‐lactones convert to aldonic acids, while the 4‐ketoaldoses are in pH‐dependent equilibrium with the corresponding *gem*‐diols.^[4]^ The LPMO regioselectivity attracts a lot of scientific attention, as it reflects how LPMOs interact with their substrate, and how the copper‐bound oxygen (or peroxide) species and the glycosidic linkage are positioned during the catalytic reaction.^[7]^

So far, singly C1 and/or C4 oxidized cello‐oligosaccharides, resulting from oxidative cleavage of cellulose, have been identified via a combination of chemically oxidized standards, high‐performance anion exchange chromatography with pulsed amperometric detection (HPAEC‐PAD) analysis, and mass spectrometric (*m/z*) data.[^7d^, ^8^] Following the same strategy, however, without including the corresponding standards, double, C1 and C4, oxidized cello‐oligosaccharides have also been suggested as LPMO products.[^7d^, ^8^] It can, however, be questioned whether the observed double oxidized products are indeed C1/C4 oxidized, or C1/C6 oxidized as suggested elsewhere,^[9]^ since both have the same *m/z* values. In this work, we analyzed different LPMO cellulose digests aided by sodium borodeuteride (NaBD_4_) reduction and hydrophilic interaction chromatography electrospray ionization negative ion mode collision‐induced dissociation mass spectrometry (HILIC‐ESI‐CID‐MS/MS^2^). We found that C4 and C1/C4 oxidizing LPMOs generated C4/C6 double oxidized cello‐oligosaccharides having a *gem*‐diol structure at C6 carbon. Our findings provide new insights into the LPMO‐mediated regioselective oxidation of cellulose, which is of high relevance for biorefinery applications.

## Results and Discussion

We first analyzed, by using HPAEC‐PAD (Figure S1), regenerated amorphous cellulose (RAC) digested with published C1/C4 oxidizing LPMOs from two different fungi: LPMO9H from *Myceliophthora thermophila* C1 (*Mt*LPMO9H)^[8b]^ and LPMO9M from *Neurospora crassa* (*Nc*LPMO9M).^[4a]^ The oligosaccharides eluting after 45 min correspond to the previously suggested double, C1 and C4, oxidized cello‐oligosaccharides.^[8b]^ The same double oxidized oligomers, eluting after 45 min, were detected in the RAC digests of the distinct C4 oxidizing *Mt*LPMO9E and *Nc*LPMO9C, though in (relatively) lower amounts, and not in C1 oxidizing *Mt*LPMO9B‐ and *Nc*LPMO9F‐RAC digests (Figure S1).

**Figure 1:**
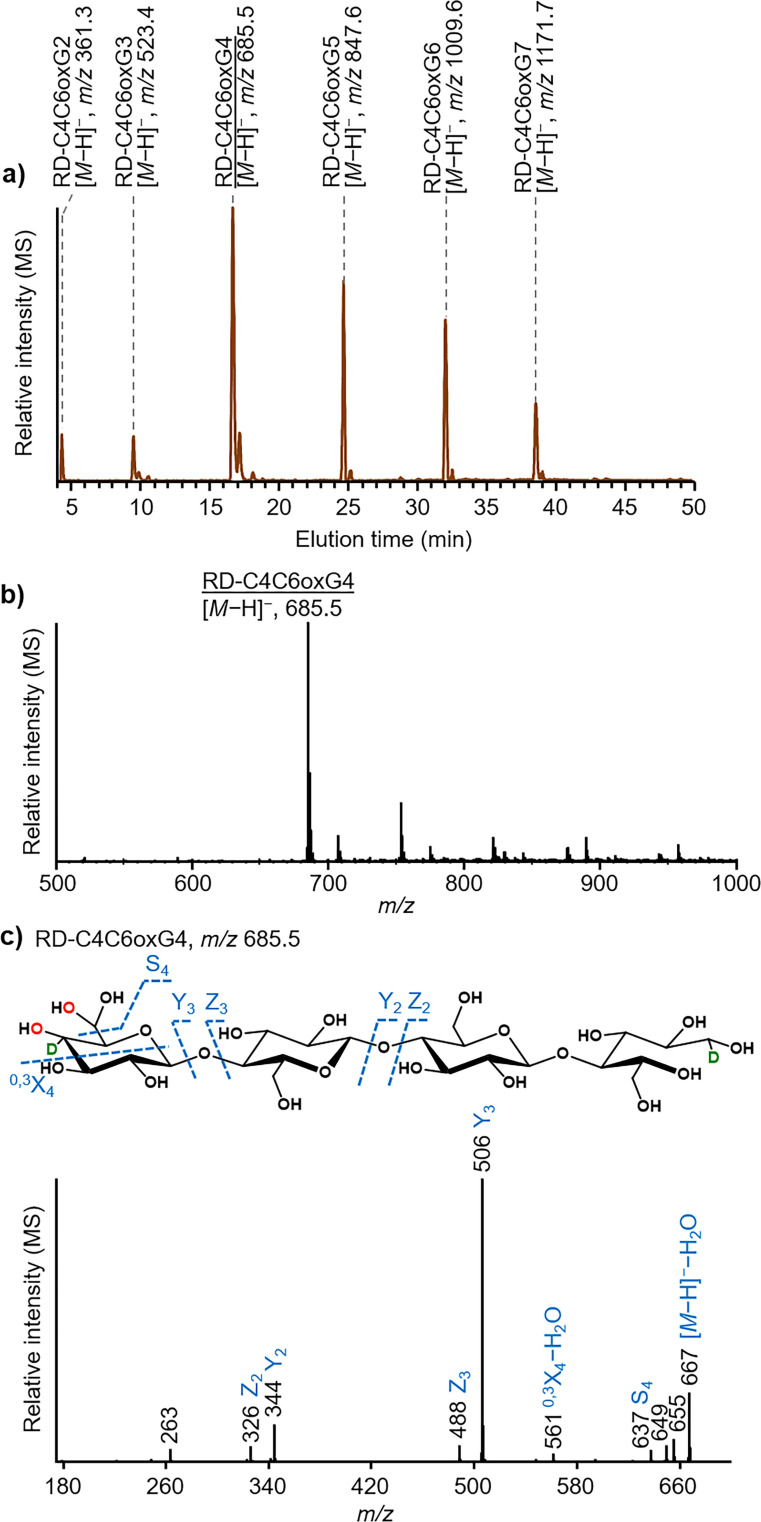
LPMO‐generated C4 and C6 double oxidized cello‐oligosaccharides after NaBD_4_ reduction in H_2_O. (a) HILIC‐ESI‐MS (negative mode; [*M*−H]^*−*^) extracted ion chromatogram of the reduced double, C4 and C6, oxidized cello‐oligosaccharides from DP2 to DP7 (RD‐C4C6oxG2–7) from the *Nc*LPMO9M‐RAC digest; similar data were obtained for *Mt*LPMO9H‐, *Nc*LPMO9C‐ and *Mt*LPMO9E‐RAC digests. (b) Representative example of an MS spectrum of the reduced double oxidized cellotetraose [RD‐C4C6oxG4, *m/z* 685.5 ([*M*−H]^*−*^)]. (c) Representative example of MS^2^ spectrum of RD‐C4C6oxG4. The oxygen atom from molecular oxygen is indicated in red and the deuterium atom (D) is indicated in green. MS^2^ spectra of RD‐C4C6OxG2–3 and RD‐C4C6OxG5–6 are shown in Figure S7. Annotation of fragments is according to the nomenclature developed by Domon and Costello.^[10]^

Next, to further understand the position of the oxidations in the double oxidized oligomers, these digests were reduced by NaBD_4_ and subsequently analyzed by HPAEC‐PAD (Figure S2) and HILIC‐ESI‐CID‐MS/MS^2^ (Figure 1 and Figure S3 in the Supporting Information).^[11]^ Reduction with NaBD_4_ reduces the reducing end of the oligosaccharides under addition of a deuterium atom (D) (see structures in Figure 1), which distinguishes this reduced end in MS^2^ fragmentation spectra from the non‐reducing end otherwise having the same *m/z* values. In addition, the C4 oxidized end becomes reduced leading to a “regular” non‐reduced end glycosyl unit, but with addition of D [*m/z* +1 compared to hydrogen atom (H)]. Both HPAEC‐PAD and HILIC‐MS showed that, besides reduced non‐oxidized (RD‐G2–6) and reduced C4 oxidized cello‐oligosaccharides (RD‐^GAL^C4oxG2–7 and RD‐^GLC^C4oxG2–7 due to the formation of galactosyl or glucosyl ends after reduction),^[11a]^ an additional series of reduced oligomers was observed (Figure S3). Reduced oligomers of this additional series were collected after HILIC analysis, and a re‐analysis by HPAEC‐PAD confirmed that these products corresponded to the additional series defined by HPAEC‐PAD, of not yet defined, possibly double, (reduced) oxidized products (Figure S4).

Further, based on *m/z* values obtained from the base peak chromatogram (Figure S3), and the extracted ion chromatogram (Figure 1a) of the HILIC‐MS/MS^2^ analysis, the additional series indicated to be a series of reduced C4/C6 double oxidized cello‐oligosaccharides (DP2–7; RD‐C4C6oxG2–7). To explain in brief, for example, for RD‐C4C6oxG4 (Figure 1b, others in Figure S5), *m/z* 685.5 ([*M*−H]^*−*^) was detected. In our previous study, we showed that (i) NaBD_4_ reduction of non‐oxidized cello‐oligosaccharides (e. g., RD‐G4, *m/z* 668.5) increases their *m/z* values (*m/z* +3) due to the reduction and insertion of one D to the reduced alditol end; (ii) NaBD_4_ reduction of C4‐oxidized cello‐oligosaccharides (e. g., RD‐C4oxG4, *m/z* 669.5) leads to an *m/z* increase of +6 compared to the non‐reduced ones (e. g., C4oxG4, *m/z* 663.5), since the C4 ketone end is reduced to −OH with one D inserted at the C4 carbon (*m/z* +3), and reduction and insertion of one D to the reduced alditol end (*m/z* +3); and (iii) (C4 oxidized) *gem*‐diol forms and C1 oxidized cello‐oligosaccharides (aldonic acid form) cannot be reduced by NaBD_4_.^[11a]^ Based on these findings, the literature‐proposed double C1/C4 oxidized cello‐oligosaccharides (e. g., C1C4oxG4, *m/z* 679.5 (C4 ketone)/ *m/z* 697.5 (C4 *gem*‐diol); Figure S6) should, after NaBD_4_ reduction, lead to an *m/z* increase of +3 in case of a C4 ketone end, or remain the same *m/z* after NaBD_4_ reduction (Figure S6). The detected *m/z* 685.5 ([*M*−H]^*−*^, Figure 1b, others in Figure S5) did not match with the *m/z* of RD‐C1C4oxG4, however, matched with a reduced form of a C4 ketone end having a C6 *gem*‐diol group (Figure 1). Corresponding *m/z* values of reduced double, C1/C4, oxidized cello‐oligosaccharides were not detected. Further, a detailed MS^2^ analysis of *m/z* 685.5 confirmed this product as a reduced form of a C4 oxidized cellotetraose having an additional (oxidized) C6 *gem*‐diol group (Figure 1 and Figure S7). In particular, *m/z* values of fragments Y_3_ and S_4_ showed the presence of an additional *gem*‐diol group on the (NaBD_4_ reduced) C4 ketone end of the oligosaccharides.

**Scheme 1:**
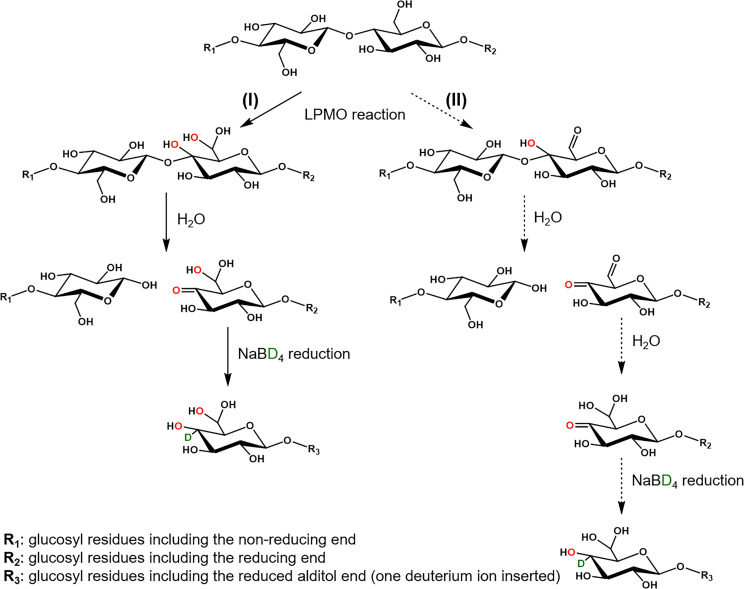
Routes for the LPMO catalyzed generation of C4/C6 double oxidized cello‐oligosaccharides. In route **I**, LPMO catalyzes the insertion of oxygen atoms at both C4 and C6 carbon atoms in the cellulose. The reaction at the C4 carbon will destabilize the glycosidic bond, leading to the bond cleavage and formation of a C4 ketone in water. After NaBD_4_ reduction, the C4 ketone is reduced to glucosyl and galactosyl residues (only one structure is used to illustrate the scheme), and the C6 *gem*‐diol remains unaltered. Route **I** is confirmed by performing the reaction and reduction in H_2_^18^O. In route **II**, the LPMO catalyzes the insertion of one oxygen atom at the C4 carbon but oxidizes the C6 carbon into a C6 aldehyde group. In water, a C4 ketone is formed, and the C6 aldehyde prefers to convert to a C6 *gem*‐diol. The subsequent reduced product is the same as in route **I**. Route **II** could not be confirmed nor disproved. The routes for generation of double, C4/C6, oxidized cello‐oligosaccharides in H_2_^18^O are presented in Figure S11. The Scheme does not distinguish whether the C4 and C6 oxidation occurs simultaneously or that C4 oxidation occurs prior to C6 oxidation.

The combined results of the NaBD_4_ reduction and the HILIC‐MS/MS^2^ analysis of the double oxidized series in the LPMO‐RAC digests, can be translated into the structure of these products prior to reduction: a C4 ketone and a C6 *gem*‐diol group in the same product (Scheme 1).

LPMOs have been shown to incorporate molecular oxygen at the C4 position, resulting in a C4 ketone, when oxygen is used as cosubstrate. The LPMO route to a C6 *gem*‐diol formation has yet to be defined. We hypothesize two routes (Scheme 1): route **I**, where the addition of a second hydroxyl group at the C6 carbon is catalyzed by the LPMO, via molecular oxygen, similar as suggested for C4 oxidation; and route **II**, where the LPMO oxidizes the C6−OH into a C6 aldehyde, which hydrates further into the C6 *gem*‐diol.^[12]^ The major difference is that in route **I** the second oxygen atom in the C6 *gem*‐diol group stems also from LPMO, while in route **II** one oxygen atom is from water.

To test the origin of the inserted oxygen atoms in the C4 ketone and C6 *gem*‐diol groups, we performed the LPMO‐RAC incubations, and the NaBD_4_ reduction, in H_2_^18^O. In these digests, reduced single C4 oxidized cello‐oligosaccharides showed a *m/z* of +2 compared to the *m/z* of corresponding RD‐C4ox generated in H_2_O (Figure S8). Corresponding MS^2^ spectra suggest the presence of an ^18^O atom (from H_2_^18^O) at the C4 carbon of the non‐reducing end. To confirm that ^18^O exchange occurred during reduction, H_2_O and H_2_^18^O digests without reduction were subjected to HILIC‐MS/MS^2^. Although the HILIC separation was poor, and the MS signal intensity of non‐reduced samples was less pronounced compared to the reduced products, we could still collect proper MS and MS^2^ spectra. From these spectra, it can be concluded that both the H_2_O and H_2_^18^O digests comprised *m/z* values consistent with ^16^O incorporation at the non‐reducing end of the C4 oxidized products. For example, for C4oxG4 *m/z* 663.5 ([*M*−H]^*−*^) was found in both H_2_O and H_2_^18^O (Figures S9 and S10).

Prior to reduction, in H_2_^18^O the *m/z* +2 values were also abundant, for example *m/z* 665.5 ([*M*−H]^*−*^). This *m/z* 665.5 can represent non‐oxidized G4 or C4oxG4 having ^18^O at the C4 of the non‐reducing end (^18O^C4oxG4), which coelutes in HILIC. They obtain the same *m/z* values that cannot be distinguished by MS and MS^2^. Nevertheless, since the *m/z* +2 values (e. g., 665.5; Figures S9 and S10) were more abundant in H_2_^18^O than in H_2_O, we suggest that the ^16^O−C4 ketone is hydrated in H_2_^18^O to a C4 *gem*‐diol (e. g., with both −^16^OH and −^18^OH), which subsequently converts back to the ^16^O− and ^18^O−C4 ketones.^[13]^ After the reduction in H_2_^18^O, mostly ^18^O−C4 ketone end‐products are observed due to the favored side of the C4 ketone over the *gem*‐diol in this equilibrium (Figure S11).

**Figure 2:**
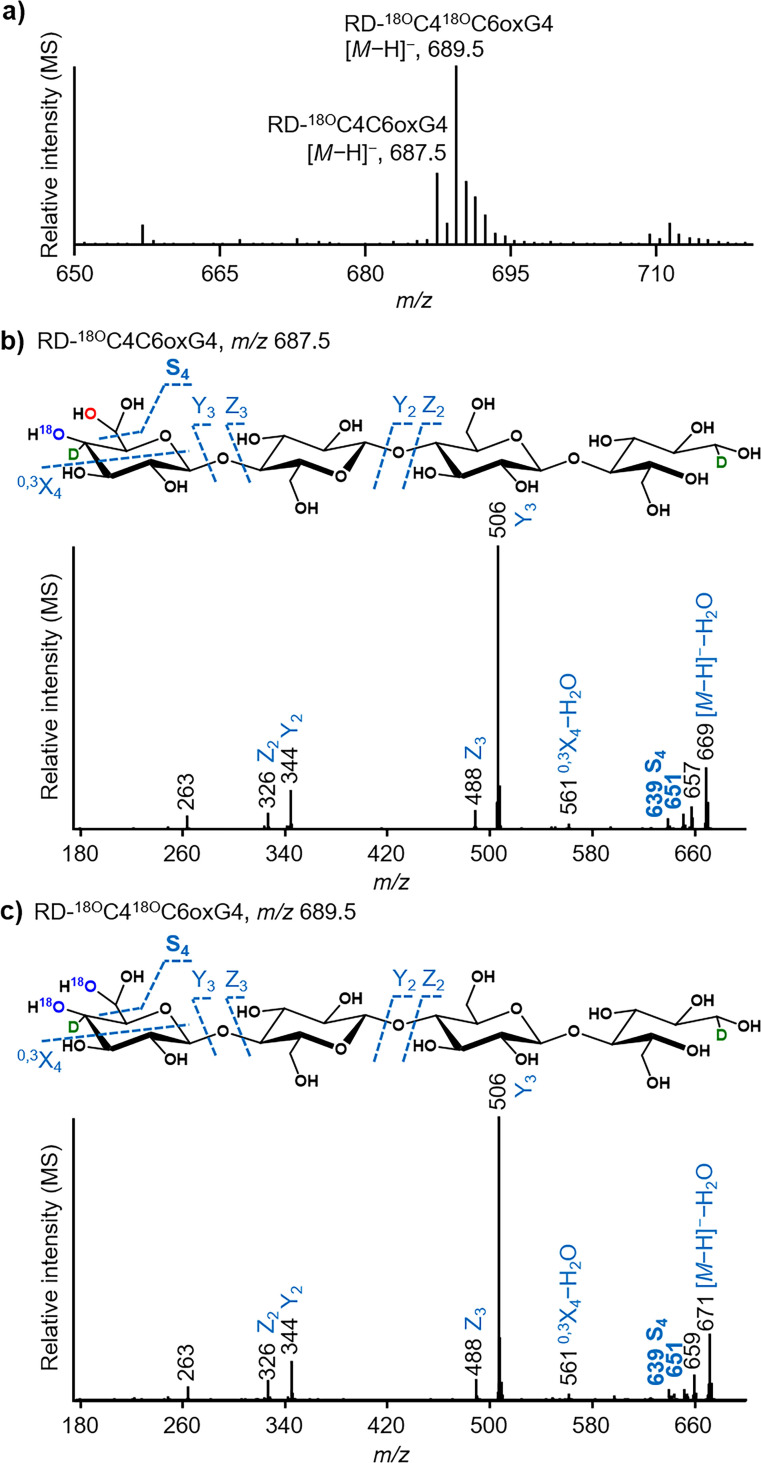
LPMO‐generated C4 and C6 double oxidized cello‐oligosaccharides after NaBD_4_ reduction in H_2_^18^O. (a) Representative example of an MS spectrum indicating the *m/z* of reduced double oxidized cellotetraose (digest and reduction in H_2_^18^O; RD‐^18O^C4C6oxG4, *m/z* 687.5 [*M*−H]^*−*^ and RD‐^18O^C4^18O^C6oxG4, *m/z* 689.5 [*M*−H]^*−*^). MS^2^ spectra of (b) RD‐^18O^C4C6oxG4 and (c) RD‐^18O^C4^18O^C6oxG4. The oxygen atom from molecular oxygen or from H_2_^18^O is indicated in red and blue, respectively. The deuterium atom (D) is indicated in green. Annotation of fragments is according to the nomenclature developed by Domon and Costello.^[10]^

With respect to the reduced double oxidized products in the H_2_^18^O digests (Figure 2a), compared to those in H_2_O (Figure 1b), the MS spectra showed a series of *m/z* +2 and of +4 values (e. g., *m/z* 687.5 [*M*−H]^*−*^; RD‐^18O^C4C6oxG4, and *m/z* 689.5 [*M*−H]^*−*^; RD‐^18O^C4^18O^C6oxG4). The corresponding MS^2^ fragmentation patterns showed that the one double oxidized series (e. g., *m/z* +2) contained ^18^O at the non‐reducing end only (Figure 2b). Notably, the *m/z* loss resulting in the MS^2^ S_4_ fragment (e. g., loss of C6 *gem*‐diol from the parental molecule) was the same in RD‐C4OxG4 and RD‐^18O^C4OxG4 (both 47), again indicating that ^16^O was found in the C6 *gem*‐diol group, even in H_2_^18^O (Figures 1c and 2b). This diagnostic observation confirmed route **I**: a second hydroxyl group in the C6 *gem*‐diol is formed via LPMO (per)oxygenation (Scheme 1 and Figure S12). The other double oxidized series (e. g., *m/z* +4) was identified, via corresponding MS^2^ spectra, to have ^18^O both at the non‐reducing end and at the C6 carbon atom (Figure 2c; RD‐^18O^C4^18O^C6oxG4), confirmed by the mass loss of 49 of the S_4_ fragment (Figure 2c). Again, as described above for ^18^O exchange at the C4 position (e. g., via *gem*‐diol formation), formation of RD‐^18O^C4^18O^C6oxG4 possibly results from RD‐^18O^C4C6oxG4, by exchange of ^16^O to ^18^O in H_2_^18^O (Figure S12). However, route **II** (Scheme 1) cannot be fully excluded as RD‐^18O^C4^18O^C6oxG4 may also form from hydration of the C6 aldehyde in H_2_^18^O (Figure S12). Prior to reduction, *m/z* values of C6 aldehyde structures were recognized (only DP3 and 4 were seemingly present; Figure S13). However, due to poor separation of non‐reduced samples and too low MS intensities of these products, the data were not conclusive, and route **II** was not confirmed nor disapproved. Oxidation to C6 aldehydes followed by hydration to C6 *gem*‐diols has been described as products from galactose oxidases and raffinose oxidases.^[14]^ These enzymes oxidize the C6 carbon of a galactosyl residue to a galactoaldehyde, followed by hydration to the C6 *gem*‐diol, hemiacetal formation and other minor modifications.^[14]^ C6 aldehyde and the two latter products were not observed to be significantly present in the reduced LPMO‐RAC digests, and products with C6 *gem*‐diol structure were predominant. These results suggested that most likely C6 *gem*‐diol is formed directly via oxygenation catalyzed by LPMOs, as demonstrated in route **I**.

We described two routes how C4/C6 double oxidized cello‐oligosaccharides formed; however, it remains to be answered whether C4/C6 double oxidized cello‐oligosaccharides were generated simultaneously with C4 oxidized ones, or that C6 oxidation was generated after C4 oxidation took place. However, the chance that C6 oxidation occurred at (early on) formed oxidized C4 ends, and *vice versa* is considered to be very low. More likely would be that in the same LPMO‐substrate complex both C4 and C6 oxidation took place. The latter is strengthened by our observation of (only) formation of C4/C6 double oxidized cello‐oligosaccharide, and C4 oxidized cello‐oligosaccharides in the LPMO reactions studied, also at earlier incubation time points (data not shown). We attempted to pick up if single C6 oxidized products were formed. Since without further cleavage of the glycosidic linkages, only C6 oxidized cellulose will not become soluble, we have investigated cellulases hydrolyzed residues of RAC and of an LPMO‐RAC digest with proton nuclear magnetic resonance (^1^H NMR) spectroscopy (Figure S14). For comparison, the soluble fraction of the LPMO‐RAC digest was also analyzed by NMR spectroscopy. Based on these NMR results, we observed basically no differences in the expected *gem*‐diol region (based on chemical shift predictions) in both RAC residues, while in the same region signals were observed for the soluble part of the LPMO digest (Figure S14). Thus, we concluded that there is no evidence that single C6 oxidation occurred. Nevertheless, further research is recommended to study the order of events of C4 and C6 double oxidation, which will shed more light on how the co‐substrate, the carbohydrate and the enzyme are oriented.

## Conclusion

In this research, we characterized, for the first time, a series of novel LPMO‐catalyzed C4/C6 double oxidized cello‐oligosaccharides by NaBD_4_ reduction and HILIC‐ESI‐CID‐MS/MS^2^. We found that C4/C6 double oxidized cello‐oligosaccharides were generated by C4 and C1/C4 oxidizing LPMOs, but not by C1 oxidizing ones. By performing LPMO‐RAC incubation and NaBD_4_ reduction in H_2_^18^O, we confirmed that the C6 *gem*‐diol structure resulted from oxygenation, though oxidation to a C6 aldehyde, followed by hydration to the C6 *gem*‐diol, could not be excluded.

## Experimental Section

### Enzymatic incubation of AA9 LPMOs with RAC

Materials, production, and purification of AA9 LPMOs used in this study are described in the Supporting Information. LPMO‐RAC incubation was based on a method reported by Sun et al.^[11a]^ 2 mg mL^−1^ of RAC was mixed with 50 mm (final concentration) ammonium acetate buffer (pH 5.0). Subsequently, 1 μm of LPMO was added to RAC mixture with and without 1 mm ascorbic acid (Asc; final concentration). Control reactions were performed in the absence of LPMO with and without Asc. All reactions (500 μL total volume) were incubated at 30 °C by using an Eppendorf Thermomixer comfort (Hamburg, Germany), placed in an almost vertical direction, at 800 rpm in duplicate. At 6 and 16 h, another 1 μm of *Mt*LPMO9H or *Mt*LPMO9E was added to their corresponding incubation tubes (in total 3 μm), while the same volume of water was added to other LPMO‐RAC incubation tubes. The reactions continued till 24 h and were stopped by immediately separating supernatants after centrifugation at 22000×*g* for 10 min at 4 °C in a benchtop centrifuge (Z 233 MK‐2, HERMLE Labortechnik GmbH, Wehingen, Germany). The resulting supernatants were collected and part of supernatants were reduced by NaBD_4_ followed by solid phase extraction (SPE) as described below.^[15]^ The non‐reduced digests were diluted five times before HPAEC‐PAD analysis. A mixture of cellobiose, cellotriose, cellotetraose, cellopentaose, and cellohexaose (1 μg mL^−1^ each) were analyzed as standards.

### Reduction of generated (oxidized) cello‐oligosaccharides with NaBD_4_ and clean‐up with SPE

Reduction with NaBD_4_ was performed according to Sun et al.^[11a]^ A clean‐up procedure for reduced digests was carried out by using SPE with Supelclean ENVI‐Carb columns (3 mL, Sigma–Aldrich) as described previously.^[11a]^ The dried reduced samples were dissolved in 60 % (*v*/*v*) acetonitrile in water. The reduced RAC digests were directly used for HILIC‐ESI‐CID‐MS/MS^2^ analysis or diluted twenty times for HPAEC‐PAD analysis.

### Enzymatic incubation and reduction in H_2_^18^O

Prior to enzymatic incubation, RAC, buffer and enzyme stock solution were prepared in H_2_^18^O. 0.7 mL of RAC suspension was thoroughly mixed with 0.7 mL of H_2_^18^O followed by centrifugation 22000×*g* for 10 min at 4 °C in a table centrifuge. Subsequently, the supernatant was discarded and H_2_^18^O was added to bring back to the same volume. These steps were repeated another four times to achieve that H_2_^18^O in RAC was >99.9 %. 50 mm (final concentration in the incubation) ammonium acetate buffer (pH 5.0) containing 1 mm Asc (final concentration in the incubation) was freshly prepared in H_2_^18^O prior to incubation. Stock solutions of *Mt*LPMO9E, *Mt*LPMO9H, *Nc*LPMO9C and *Nc*LPMO9M were concentrated ten times by using Amicon Ultra‐0.5 centrifugal filter units (Merck Millipore, U.S.A.) with a 3 kDa cutoff. Afterwards, 50 mM ammonium acetate buffer (pH 5.0) in H_2_^18^O was added to bring back to the same volume. These cycles were repeated another four times to achieve that H_2_^18^O in enzyme stock solution was >99.9 %. The enzymatic incubation with RAC was performed as described above. The reduction was carried out by using NaBD_4_ in H_2_^18^O. The SPE procedures were performed as described above. The dried reduced samples were dissolved in 60 % (*v*/*v*) acetonitrile in H_2_^18^O just prior to analysis.

### HPAEC‐PAD analysis for profiling oligosaccharides

Samples and standards were analyzed by a HPAEC‐PAD with an ICS‐5000 system (Dionex, Sunnyvale, California, U.S.A.) equipped with a CarboPac PA‐1 column (2 mm ID × 250 mm; Dionex) in combination with a CarboPac PA guard column (2 mm ID × 50 mm; Dionex). Two mobile phases were (A) 0.1 m NaOH and (B) 1 m NaOAc in 0.1 m NaOH and other setting have been described previously.[^11a^, ^16^] Non‐reduced samples and standards were analyzed with a 92.5 min elution program: 0–45 min, linear gradient 0–25 % B; 45–67.5 min, linear gradient 25–50 % B; 67.5–77.5 min isocratic gradient 100 % B, and 77.5–92.5 min isocratic gradient 0 % B. The elution program for analysis of reduced samples and standards has been described previously.[^11a^, ^16^] Chromatographic data were processed by using Chromeleon 7.2.10 software (Thermo Fisher Scientific, Waltham, Massachusetts, U.S.A.).

### HILIC‐ESI‐CID‐MS/MS^2^ for structural elucidation of reduced cello‐oligosaccharides

The reduced RAC digests were analyzed by HILIC‐ESI‐CID‐MS/MS^2^ on a Vanquish UHPLC system (Thermo Fisher Scientific) coupled to an LTQ Velos Pro mass spectrometer (Thermo Fisher Scientific). The UHPLC and MS (negative ion mode) settings, column and mobile phases have been described previously.^[11a]^ The elution program was also described previously.^[11b]^ MS^2^ was performed under dependent scan mode as described previously with the only modification of Activation Q set to 0.25.^[11a]^ MS data were processed by using Xcalibur 2.2 software (Thermo Fisher Scientific). All chemical structures used were created by using ChemDraw 18.0.0.231 (PerkinElmer, Waltham, Massachusetts, U.S.A.).

## Conflict of interest

The authors declare no conflict of interest.

## Supporting information

As a service to our authors and readers, this journal provides supporting information supplied by the authors. Such materials are peer reviewed and may be re‐organized for online delivery, but are not copy‐edited or typeset. Technical support issues arising from supporting information (other than missing files) should be addressed to the authors.

Supporting Information
