# Supplementary material for: Regioselective C4 and C6 Double Oxidation of Cellulose by Lytic Polysaccharide Monooxygenases
Source: ChemSusChem. 2021 Dec 18;15(2):e202102203. doi: 10.1002/cssc.202102203 (PMC9299857; doi:10.1002/cssc.202102203)
Supplement: Supplementary file 1 — Supporting Information [file CSSC-15-0-s001.pdf]

ChemSusChem

Supporting Information

Regioselective C4 and C6 Double Oxidation of Cellulose by Lytic Polysaccharide Monooxygenases

Peicheng Sun, Christophe V. F. P. Laurent, Vincent J. P. Boerkamp, Gijs van Erven, Roland Ludwig, Willem J. H. van Berkel, and Mirjam A. Kabel*© 2021 The Authors. ChemSusChem published by Wiley-VCH GmbH. This is an open access article under the terms of the Creative Commons Attribution License, which permits use, distribution and reproduction in any medium, provided the original work is properly cited.

Supporting Information content:

Materials

Production and purification of AA9 LPMOs

Proton nuclear magnetic resonance spectroscopy analysis of LPMO-RAC residue and supernatant

Figure S1. HPAEC chromatograms of different LPMO-RAC digests

Figure S2. HPAEC chromatograms of different reduced LPMO-RAC digests

Figure S3. HILIC-ESI-MS base peak chromatograms of different reduced LPMO-RAC digests

Figure S4. HPAEC chromatograms of RD-C4C6oxG2 to RD-C4C6oxG6 collected from HILIC separation

Figure S5. HILIC-ESI-MS of RD-C4C6oxG2–3 and RD-C4C6oxG5–6

Figure S6. HILIC-ESI-MS of RD-C4C6oxG2–3 and RD-C4C6oxG5–6

Figure S7. Previously suggested structures of C1/C4 double oxidized cello-oligosaccharides before and after $NaBD_4$ reduction

Figure S8. HILIC-ESI-MS and CID-MS^2 of RD-C4oxG4 and RD-^{18O}C4oxG4

Figure S9. HILIC-ESI-MS and CID-MS^2 of C4oxG4 and G4 formed in H_2O

Figure S10. HILIC-ESI-MS and CID-MS^2 of C4oxG4, G4, and ^{18O}C4oxG4 in $H_2{}^{18}O$

Figure S11. Proposed route of formation of RD-^{18O}C4ox

Figure S12. Proposed route of formation of RD-^{18O}C4C6ox and RD-^{18O}C4^{18O}C6ox

Figure S13. HILIC-ESI-MS of possible C4C6oxG3 and C4C6oxG4 having C6 aldehyde structure

Figure S14. ^{1}H NMR spectra of cellopentaose, LPMO-RAC supernatant, cellulase hydrolyzed untreated RAC and LPMO-RAC residue

References

Materials

Cellobiose, cellotriose, cellotetraose, cellopentaose and cellohexaose were purchased from Megazyme (Bray, Ireland). Regenerated amorphous cellulose (RAC) was prepared from AVI (Avicel® PH-101, Sigma-Aldrich, St. Louis, Missouri, USA) as described previously.[1] Ascorbic acid (Asc) was purchased from VWR International (Radnor, Pennsylvania, USA) and sodium borodeuteride ($NaBD_4$) was purchased from Sigma-Aldrich. Water ($H_2{}^{16}O$) used was produced via a Milli-Q system (Millipore, Molsheim, France). $H_2{}^{18}O$ (GMP grade, ^{18}O >97%) was purchased from ABX advanced biochemical compounds-Biomedizinische Forschungsreagenzien GmbH (Radeberg, Germany). Deuterated water (D_2O, D >99%) was purchased from Eurisotop (SaintAubin, France). Celluclast® 1.5L was purchased from Novozymes (Bagsværd, Denmark) and fractionated before use as described previously.[2] Other chemicals were purchased from either Sigma-Aldrich or VWR International.

Production and purification of AA9 LPMOs

The genes encoding *Mt*LPMO9B (MYCTH_80312; UniProt ID, G2QCJ3), *Mt*LPMO9E (MTCTH_79765, UniProt ID: G2Q7A5) and *Mt*LPMO9H (MYCTH_46583, UniProt ID: G2Q9T3) were homologously expressed in a low protease/low (hemi-) cellulose producing *Myceliophthora thermophila* C1 strain (IFF Nutrition & Biosciences, Leiden, The Netherlands), as described elsewhere.[3] Purification of the resulting *Mt*LPMO enzymes has been described previously.[2, 4] Production and purification of *Nc*LPMO9C, *Nc*LPMO9F and *Nc*LPMO9M have been described elsewhere.[5]

Proton nuclear magnetic resonance spectroscopy analysis of LPMO-RAC residue and supernatant

Prior to proton nuclear magnetic resonance (^1H NMR) spectroscopy analysis, the insoluble residue from the *Mt*LPMO9H-RAC digest was treated with the cellulase cocktail Celluclast® 1.5L (48.58 mg protein/mL) in order to solubilize the residue. Hereto, 5 µL of ten times diluted (with D_2O) Celluclast® 1.5L was added to repeatedly D_2O washed untreated RAC and to *Mt*LPMO9H-digested RAC residue suspension (400 µL; ~1 mg dry matter content; washed five times with 1 mL D_2O). A control sample was prepared with only the 5 µL of ten times diluted (with D_2O) cellulase cocktail Celluclast® 1.5L in 400 µL D_2O. These three samples were incubated at 50 °C in an Eppendorf Thermomixer® comfort (Hamburg, Germany) rotating at 800 rpm. After every hour of incubation, another 5 µL of ten times diluted Celluclast® 1.5L was added to the corresponding samples, with 7 additions in total. Afterwards, samples were immediately transferred to 5 mm NMR tubes and stored at 4 °C before NMR analysis. After the extensive cellulase treatment, still some insoluble material was observed for both RAC samples. For comparison, supernatants from the *Mt*LPMO9H-RAC digest and from the incubation of RAC with only Asc were prepared for NMR analysis. These supernatants (1 mL each) were dried under nitrogen flow, redissolved in 450 µL D_2O, and transferred to 5 mm NMR tubes. 1 mg of the standard cellopentaose was dissolved in 450 µL D_2O. For NMR measurement, the D_2O contained a trace amount of methanol which was used as internal reference (δ_H 3.34 ppm). Spectra were recorded on a Bruker Avance HD 700 MHz NMR spectrometer (Bruker BioSpin, Switzerland) equipped with a 5 mm BBI-probe. The internal temperature of the probe was set to 298 K. In total 8192 scans were collected using an FID size

of 64k, a spectral width of 20 ppm, and offset of 4.7 ppm (δ_H 14.7 to -5.3 ppm). The number of scans was set this high to be able to detect minor signals as well. The relaxation time was set to 2 s, the acquisition time to 2.3 s, the 90° pulse length was automatically determined (~8 μs), and the receiver gain was maximized. Water suppression was achieved using the standard Bruker pulse program "noesygppr1d", where the water signal is pre-saturated during the relaxation delay, mixing time, and spoil gradient. The same acquisition parameters as above were used for the standard cellopentaose, except for the number of scans which was reduced to 128. The data was processed in MestReNova v14.1 (Mestrelab Research, S.L., Santiago de Compostela, Spain). For the Fourier transform, an exponential window function was applied using a line broadening factor of 0.3 Hz.

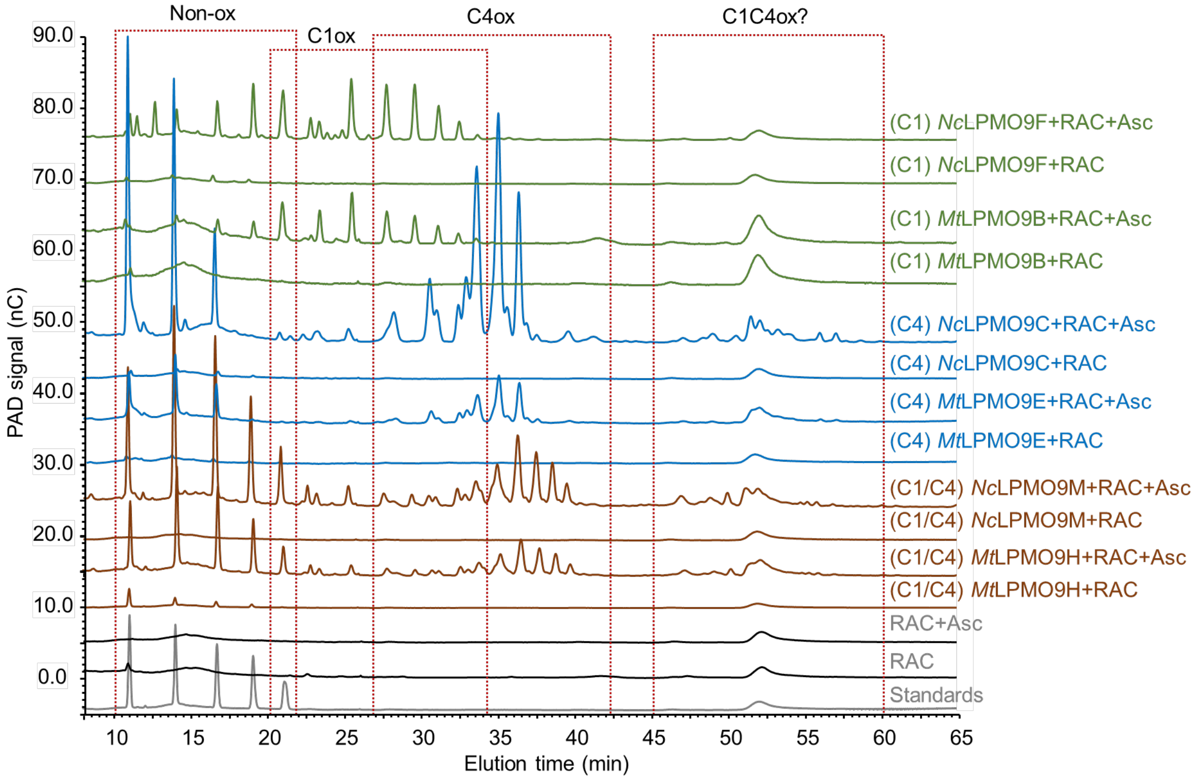

Figure S1. HPAEC chromatograms of LPMO-RAC digests and control samples. *Mt*LPMO9H and *Nc*LPMO9M were previously characterized as C1/C4 oxidizing LPMOs.[6] *Mt*LPMO9E and *Nc*LPMO9C were previously characterized as C4 oxidizing LPMOs.[4, 7] *Mt*LPMO9B and *Nc*LPMO9F were previously characterized as C1 oxidizing LPMOs.[5a, 8] Elution time regions of non-, C1, C4 and C1/C4 double oxidized cello-oligosaccharides are indicated as non-ox, C1ox, C4ox and C1C4ox, respectively. A standard mixture of cellobiose, cellotriose, cellotetraose, cellopentaose and cellohexaose (standards) is also shown. The assignment of C1C4ox has been suggested previously.[6a, 9] Ascorbic acid (Asc) was the reducing agent for the LPMO reactions.

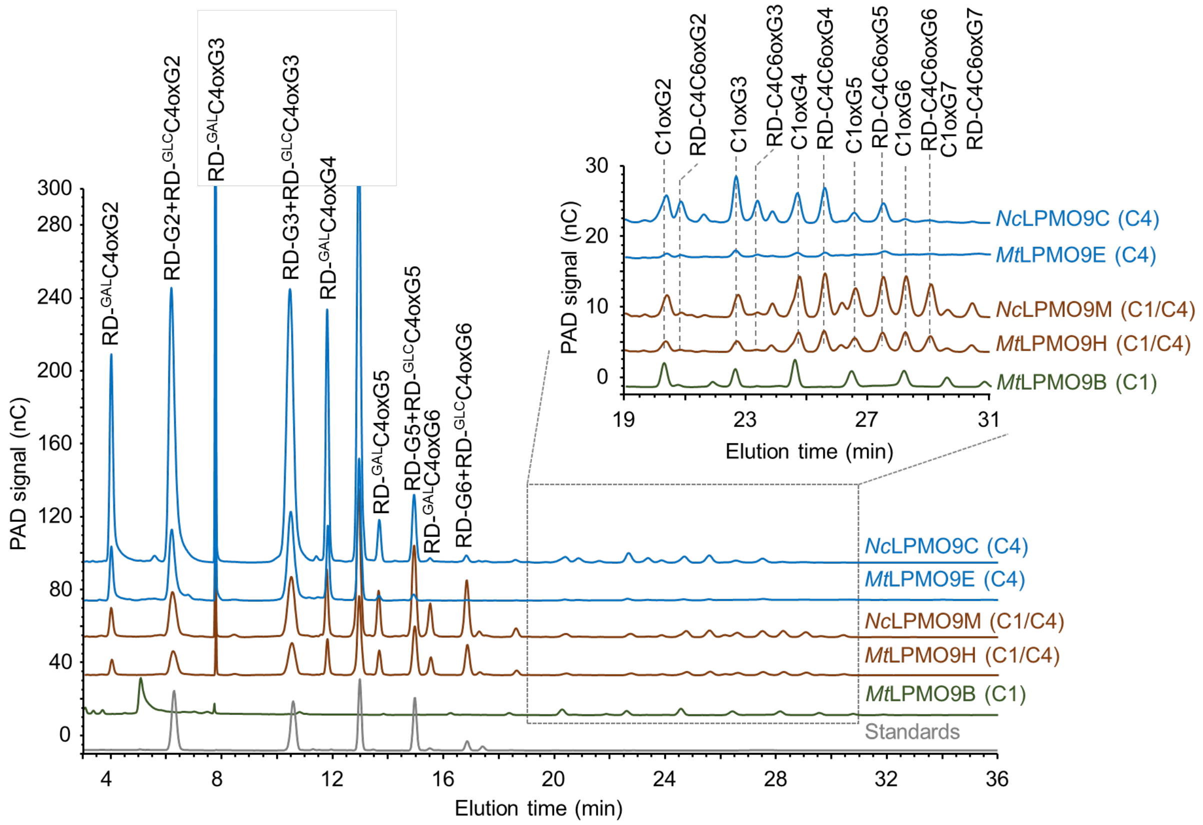

Figure S2. HPAEC chromatograms of reduced *Mt*LPMO9H-, *Nc*LPMO9M-, *Mt*LPMO9E- and *Nc*LPMO9C-RAC digests. After reduction, non-oxidized cello-oligosaccharides are converted to their alditol forms (RD-Gn), C4 oxidized ones are converted to their alditol forms with either glucosyl or galactosyl residues at the non-reducing end (RD-^{GLC}C4oxGn and RD-^{GAL}C4oxGn)[4], and C4C6 double oxidized ones are converted to their corresponding alditol forms, which are described in the main text (RD-C4C6oxGn). RD-^{GLC}C4oxGn and RD-^{GAL}C4oxGn were annotated based on $NaBD_4$ reduction of i) cello-oligosaccharide standard mixture (cellobiose, cellotriose, cellotetraose, cellopentaose and cellohexaose) (RD-G2–RD-G6, in grey) and ii) β-glucosidase hydrolyzed C4 oxidizing LPMO-RAC digest, as described previously.[4] *Mt*LPMO9B-RAC digest is also included for the annotation of C1 oxidized cello-oligosaccharides (C1oxGn). A standard mixture of cellobiose, cellotriose, cellotetraose, cellopentaose and cellohexaose after reduction (standards) is also shown. All digests shown were prepared with ascorbic acid as the reducing agent for the LPMO reactions.

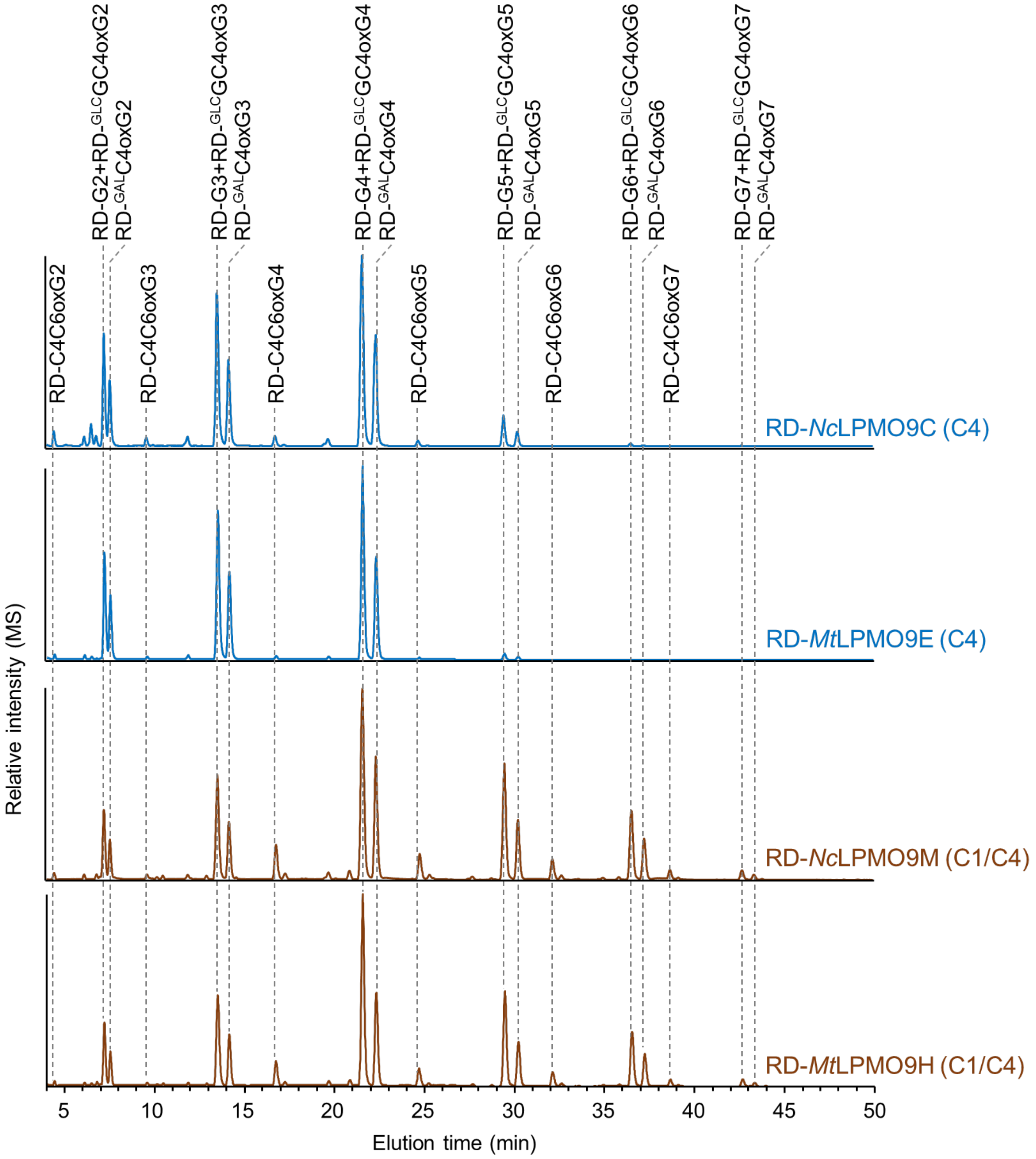

Figure S3. HILIC-ESI-MS base peak chromatograms of reduced *Mt*LPMO9H-, *Nc*LPMO9M-, *Mt*LPMO9E- and *Nc*LPMO9C-RAC digests (RD-*Mt*LPMO9H, RD-*Nc*LPMO9M, RD-*Mt*LPMO9E and RD-*Nc*LPMO9C). All digests shown were prepared with ascorbic acid as the reducing agent for the LPMO reactions. After reduction, C4/C6 double oxidized cello-oligosaccharides DP2–7 (RD-C4C6oxG2 to RD-C4C6oxG7) were identified in addition to non- (RD-G2 to RD-G7) and C4 oxidized ones (RD-^{GLC}C6oxG2 to RD-^{GLC}C4oxG7; RD-^{GAL}C6oxG2 to RD-^{GAL}C4oxG7).

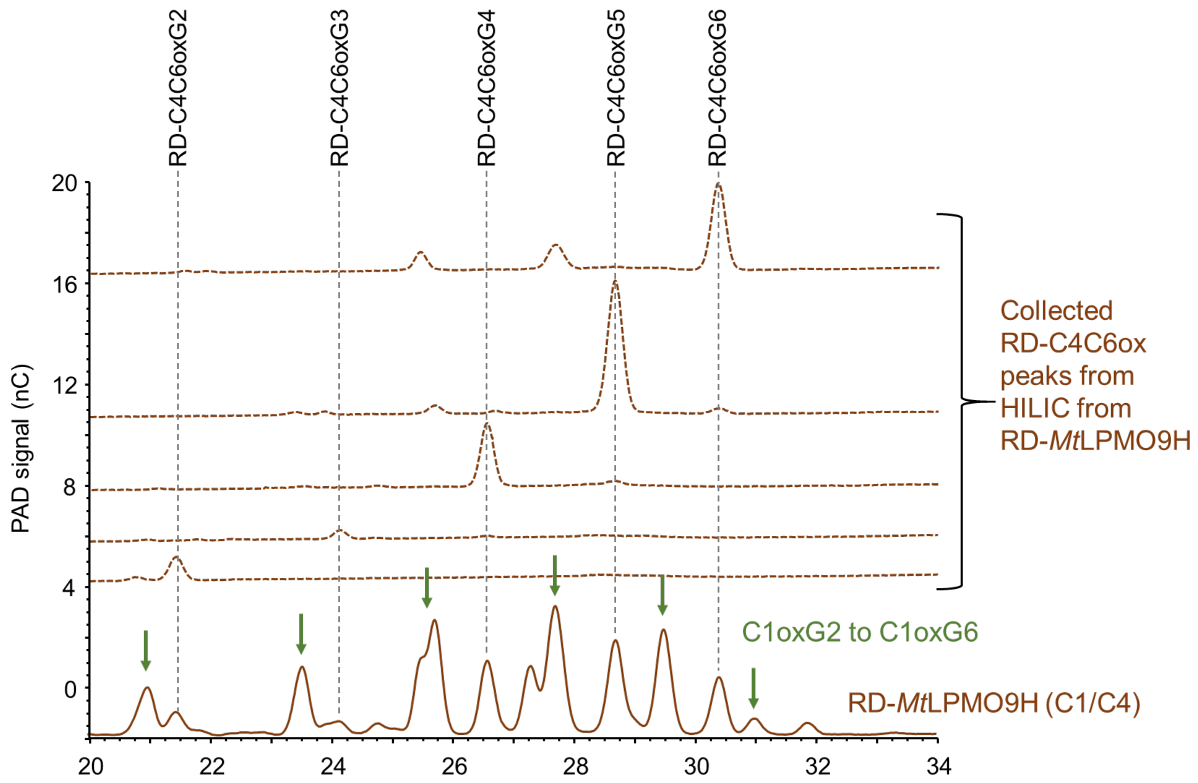

Figure S4. HPAEC chromatograms of RD-C4C6oxG2 to RD-C4C6oxG6 collected from HILIC separation prior to MS analysis. Green arrows indicate the C1oxG2 to C1oxG6.

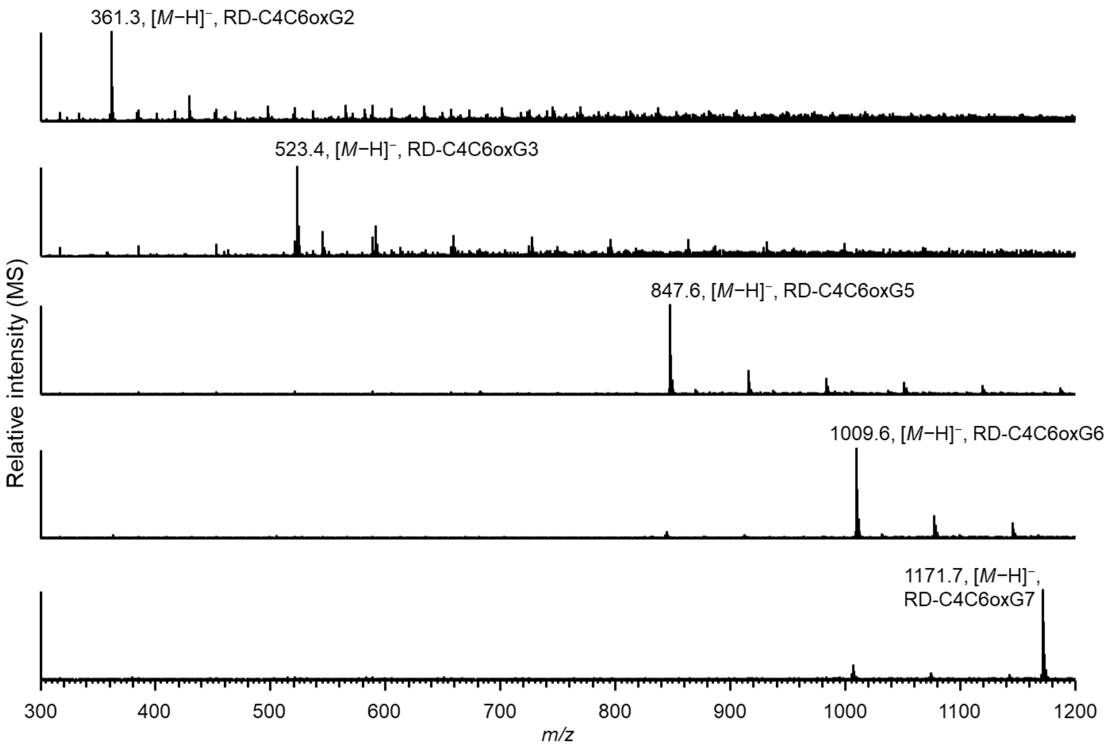

Figure S5. HILIC-ESI-MS (negative mode; [*M*–H]$^{-}$) of RD-C4C6oxG2 (*m/z* 361.3), RD-C4C6oxG3 (*m/z* 524.3), RD-C4C6oxG5 (*m/z* 847.6), RD-C4C6oxG6 (*m/z* 1009.6) and RD-C4C6oxG7 (*m/z* 1171.7). HILIC-ESI-MS of RD-C4C6oxG4 is shown in Figure 1.

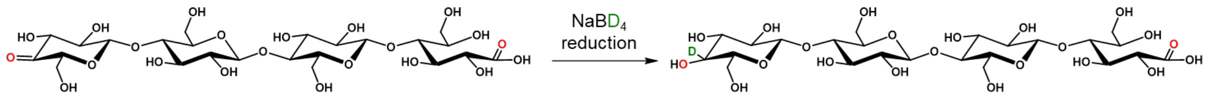

C1C4oxG4, $[M\text{–}H]^-$, *m/z* 679.5

RD-C1C4oxG4, $[M\text{–}H]^-$, *m/z* 682.5

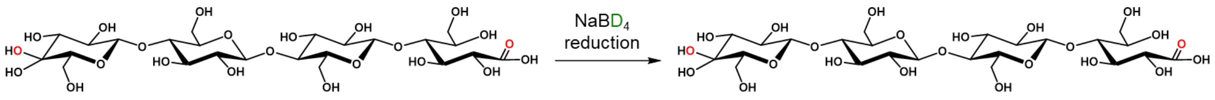

C1C4oxG4, $[M\text{–}H]^-$, *m/z* 697.5

RD-C1C4oxG4, $[M\text{–}H]^-$, *m/z* 697.5

Figure S6. Previously suggested two forms of C1/C4 double oxidized cello-oligosaccharides (e.g., DP4, C1C4oxG4). *M/z* values here are calculated and based on negative ion mode ($[M\text{–}H]^-$). The C4 ketone form of C1C4oxGn has a *m/z* difference of 3 before and after $NaBD_4$ reduction (e.g., C1C4oxG4, *m/z* 679.5 and RD-C1C4oxG4, *m/z* 682.5). If the C1C4ox is in the C4 gem-diol form, the *m/z* remains the same after reduction, because the C1 aldonic acid and C4 gem-diol structure cannot be reduced. The oxygen atom from molecular oxygen is shown in red.

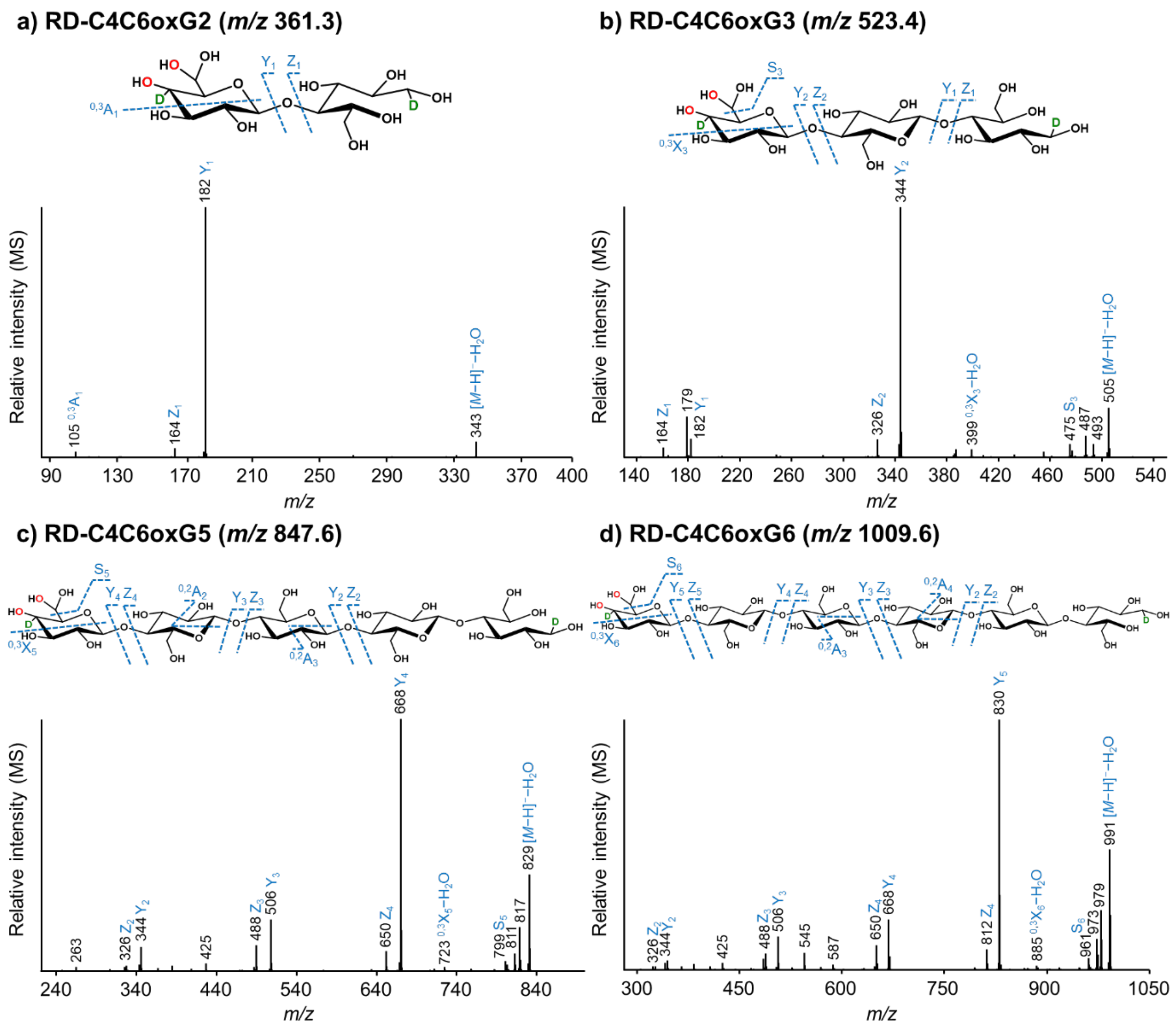

Figure S7. Negative ion mode CID-MS^2 fragmentation patterns of reduced C4 and C6 double oxidized cello-oligosaccharides (a) DP2 (RD-C4C6oxG2, *m/z* 361.3), (b) DP3 (RD-C4C6oxG3, *m/z* 523.4), (c) DP5 (RD-C4C6oxG5, *m/z* 847.6) and (d) DP6 (RD-C4C6oxG6, *m/z* 1009.6) in the *Mt*LPMO9H-RAC digest. Comparable MS^2 fragmentation patterns were also found in *Nc*LPMO9M-, *Mt*LPMO9E- and *Nc*LPMO9C-RAC digests. The oxygen atom from molecular oxygen is shown in red and the deuterium atom is shown in green. Annotation of fragments is according to the nomenclature developed by Domon and Costello.[10]

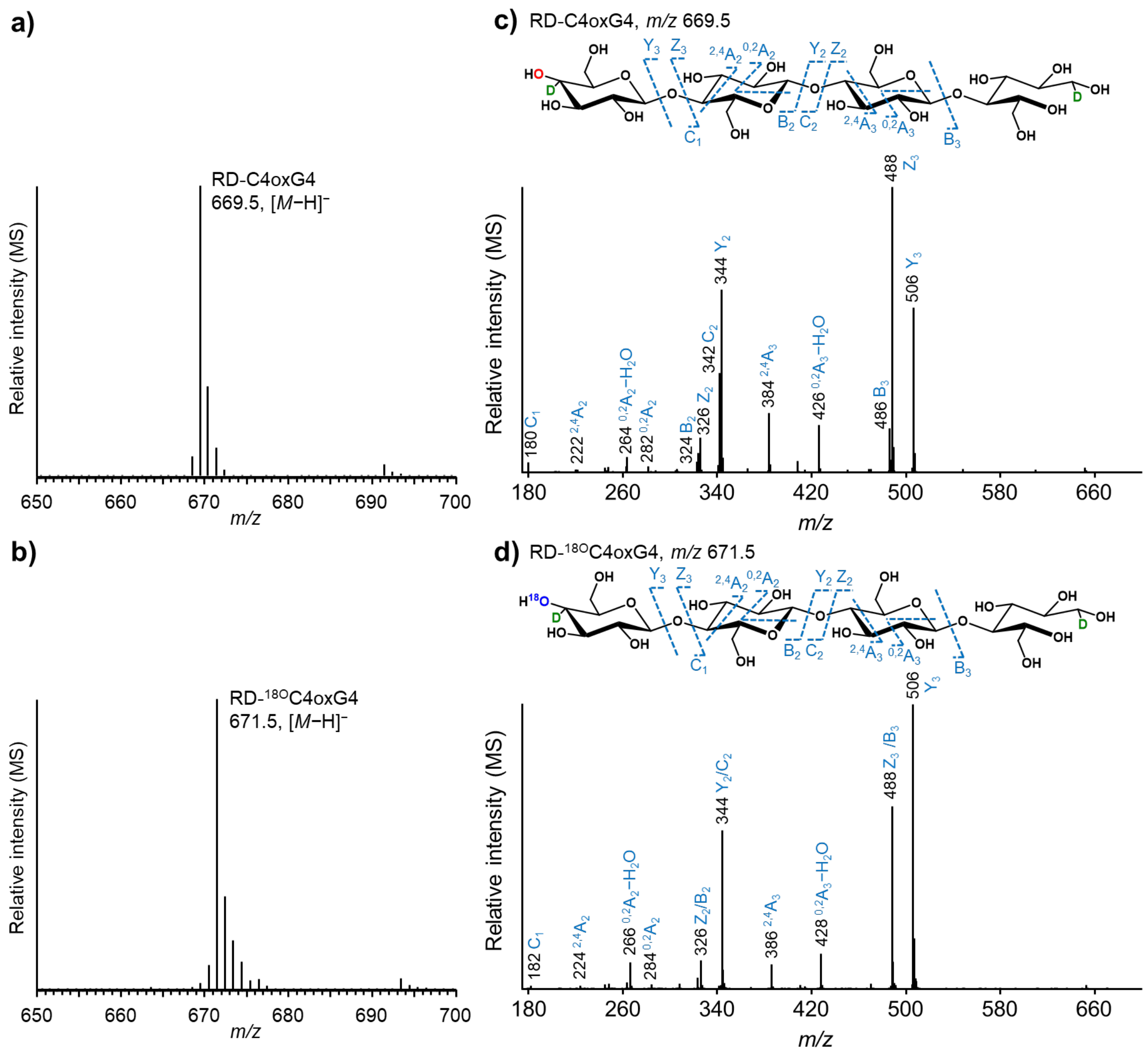

Figure S8. HILIC-ESI-MS of RD-C4oxG4 (*m/z* 669.5, a) and RD-^{18}OC4oxG4 (*m/z* 671.5, b). RD-C4oxG4 and RD-^{18}OC4oxG4 are examples of reduced C4 oxidized cello-oligosaccharides generated in H_2O and $H_2{}^{18}O$, respectively. Negative ion mode CID-MS^2 fragmentation patterns of RD-C4oxG4 (*m/z* 669.5, c) and RD-^{18}OC4oxG4 (*m/z* 671.5, d). The oxygen atom from molecular oxygen or from $H_2{}^{18}O$ is indicated in red and blue, respectively. The oxygen atom comes from molecular oxygen (red) and there is slow exchange to ^{18}O (blue) in $H_2{}^{18}O$. The deuterium atom is indicated in green. Annotation of fragments is according to the nomenclature developed by Domon and Costello.[10]

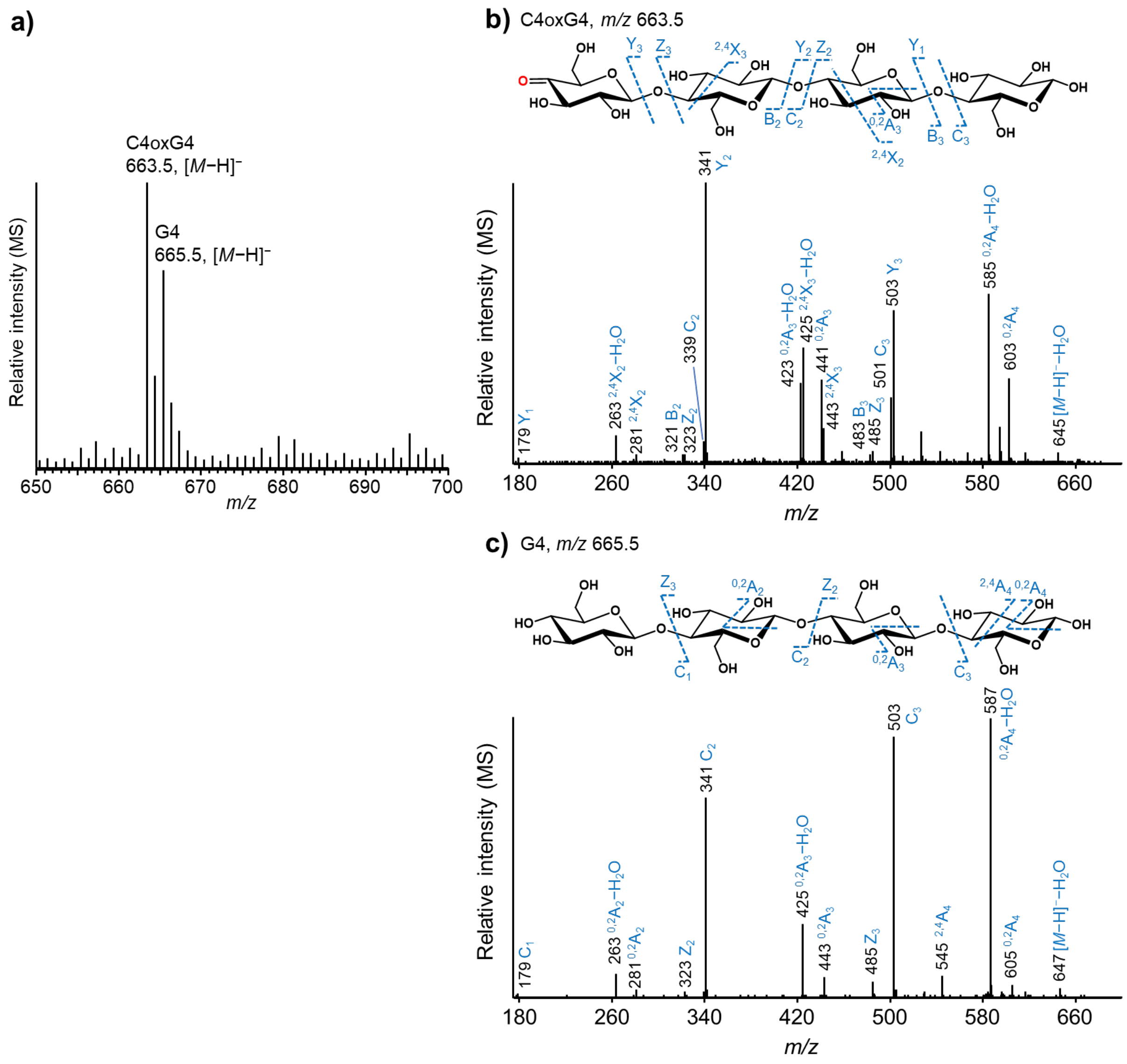

Figure S9. HILIC-ESI-MS of C4oxG4 (*m/z* 663.5) and G4 (*m/z* 665.5) (a) generated in H_2O before reduction. Negative ion mode CID-MS^2 fragmentation patterns of C4oxG4 (*m/z* 663.5, b) and G4 (*m/z* 665.5, c). These patterns were comparable as published previously.[4] The oxygen atom from molecular oxygen is indicated in red. Annotation of fragments is according to the nomenclature developed by Domon and Costello.[10]

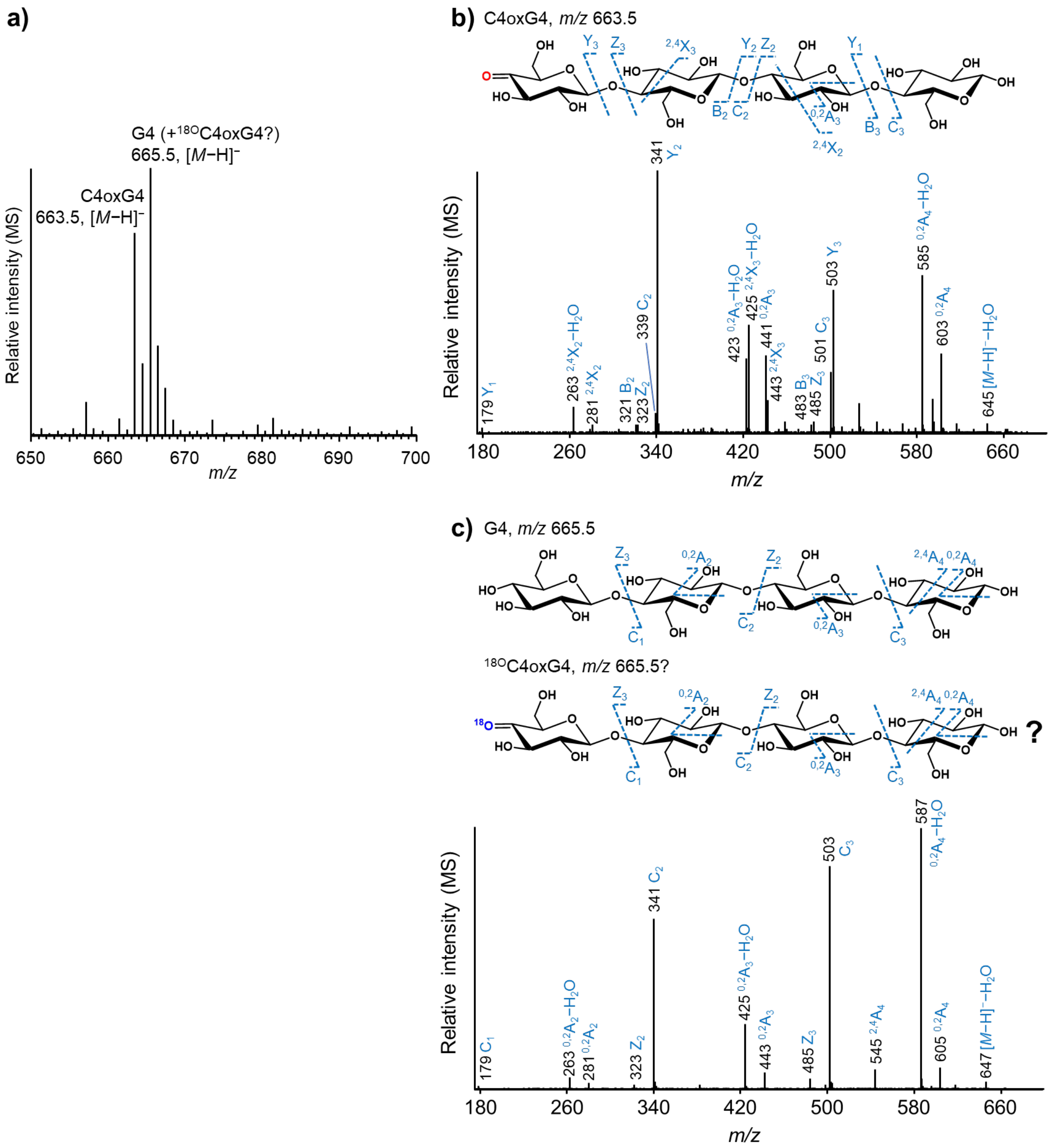

Figure S10. HILIC-ESI-MS of C4oxG4 (*m/z* 663.5) and G4 (*m/z* 665.5) (a) generated in $H_2{}^{18}O$ before reduction. ^{18}OC4OxG4 could also be present due to the increased relative intensity of *m/z* 665.5 that is the same as G4. Negative ion mode CID-MS^2 fragmentation patterns of C4oxG4 (*m/z* 663.5, b) and G4 (*m/z* 665.5, c). These patterns were comparable as published previously.[4] The oxygen atom from molecular oxygen or from $H_2{}^{18}O$ is indicated in red and blue, respectively. Annotation of fragments is according to the nomenclature developed by Domon and Costello.[10]

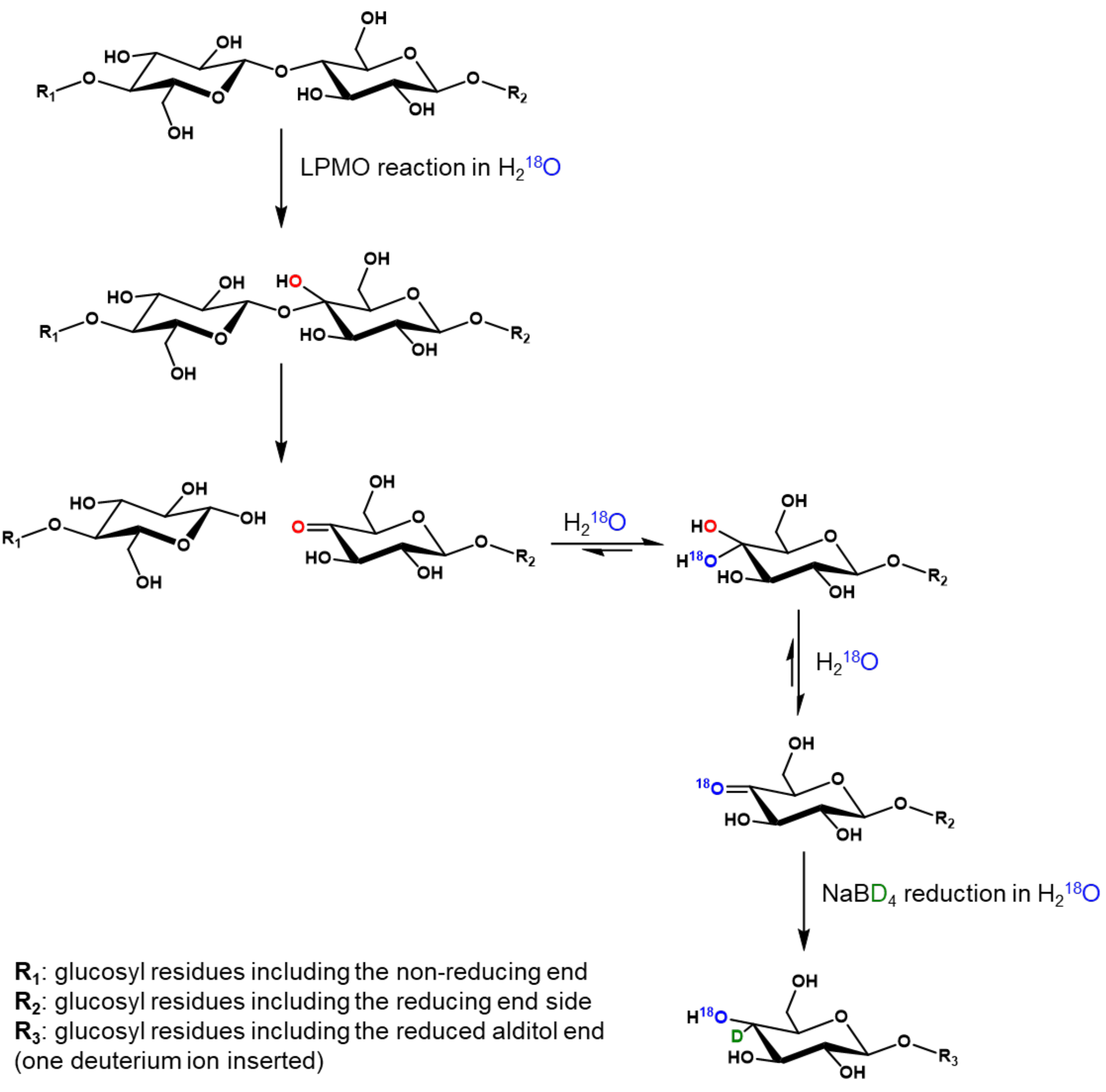

Figure S11. Proposed route of formation of RD-^{18O}C4ox. LPMO catalyzes the hydroxylation at the C4 carbon position, which destabilizes the glycosidic bond, leading to the bond cleavage and generation of C4 oxidized cello-oligosaccharides in water (C4 ketone). Here, the oxygen atom (in red) is from dioxygen (O_2) even the reaction is in $H_2{}^{18}O$. Due to the equilibrium between C4 ketones and C4 gem-diols, C4 ketones with ^{16}O (red) convert to C4 gem-diol with one ^{16}O and one ^{18}O (blue) in $H_2{}^{18}O$. This C4 gem-diol further converts back to C4 ketone but with only ^{18}O at the C4 carbon position. The $NaBD_4$ reduction in $H_2{}^{18}O$ initially consumes ^{16}O-C4 ketones and ^{18}O-C4 ketones. However, only ^{18}O-C4 ketones are generated, leading to the presence of predominant of RD-^{18O}C4ox. Note that RD-^{18O}C4ox has either glucosyl or galactosyl ends. To simplify the scheme, only one structure is used here.

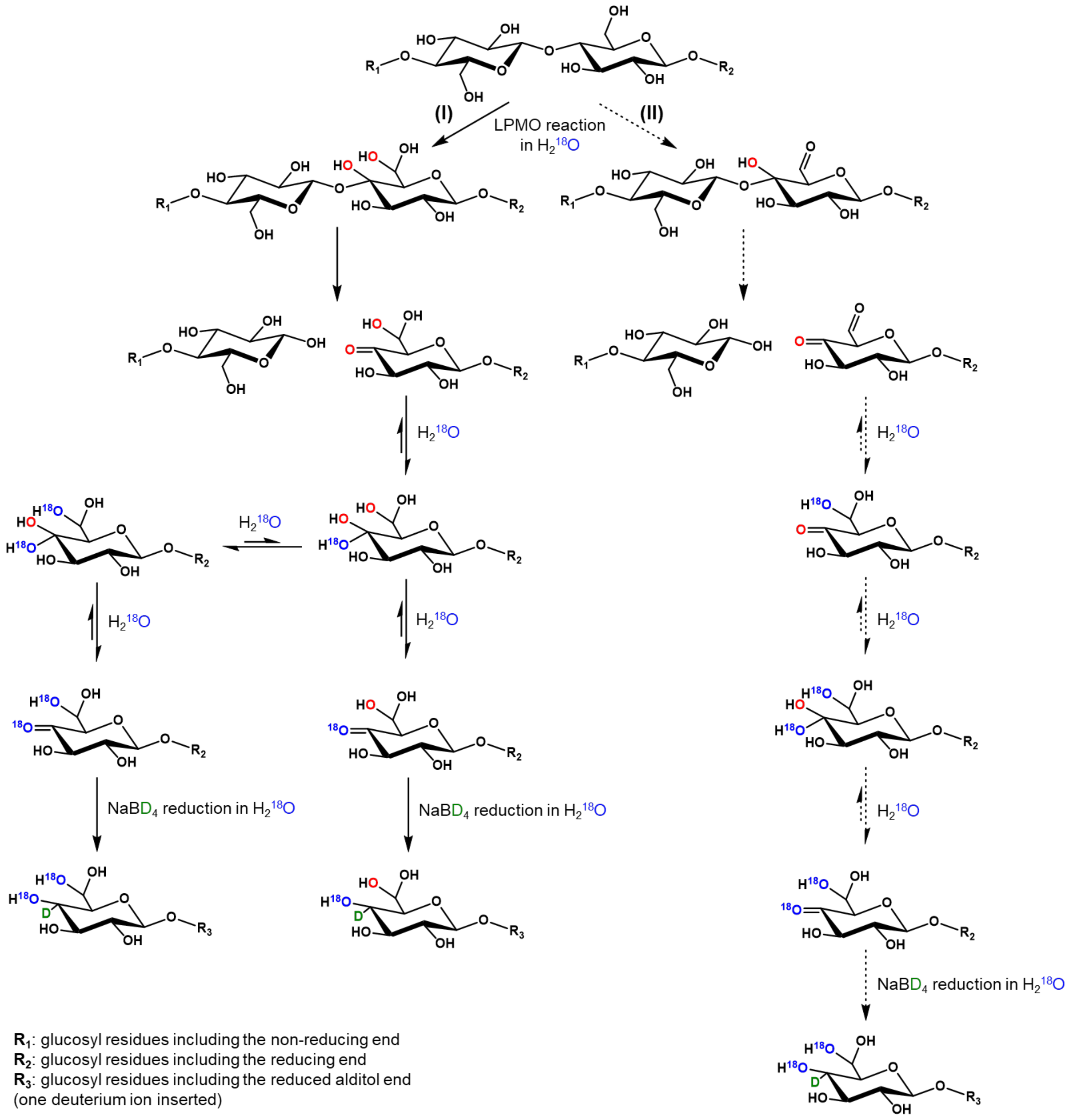

Figure S12. Proposed routes of formation of RD-^{18}OC4C6ox and RD-^{18}OC4^{18}OC6ox. LPMO catalyzes the hydroxylation at the C4 carbon position, which destabilizes the glycosidic bond, leading to the bond cleavage and generation of C4 oxidized cello-oligosaccharides in water (C4 ketone). Here, the oxygen atom (in red) is from dioxygen (O_2) even the reaction is in $H_2{}^{18}O$. In route I, C6 gem-diol is formed by LPMO reaction with one oxygen atom originating from dioxygen (O_2). The conversion of C4-ketone after $NaBD_4$ reduction in $H_2{}^{18}O$ is described in Figure S11 caption. C6 gem-diol remains after reduction as it cannot be reduced by $NaBD_4$ (RD-^{18}OC4C6ox). In route II, the formation of C4 ketone is the same as in route (a). The C6 carbon is oxidized by LPMO reaction to C6 aldehyde, which is subsequently hydrated to C6 gem-diol. After reduction, RD-^{18}OC4^{18}OC6ox is formed as described in route I. As RD-^{18}OC4^{18}OC6ox can be derived from RD-^{18}OC4C6ox in $H_2{}^{18}O$, the two-step formation of C6 gem-diol (route II) needs to be further validated.

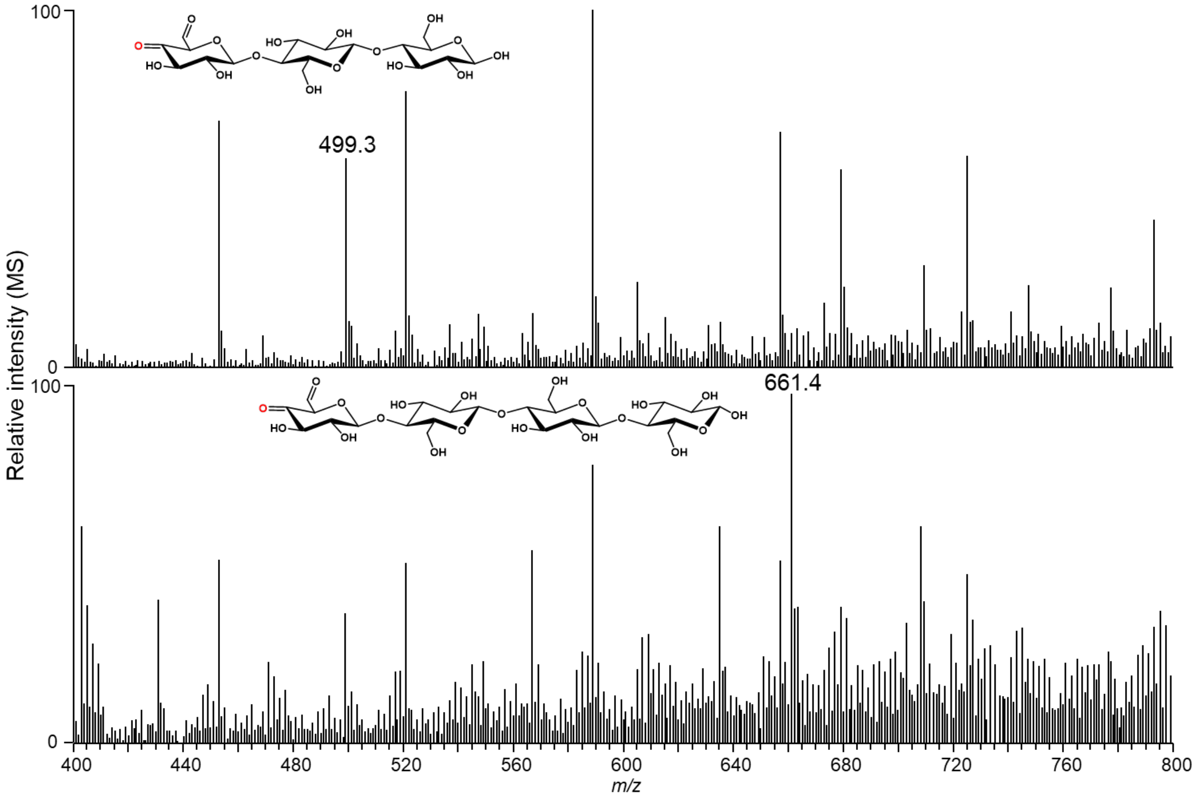

Figure S13. HILIC-ESI-MS (negative mode; [*M*–H]⁻) of possible C4C6oxG3 (*m/z* 499.3) and C4C6oxG4 (*m/z* 661.4) having C6 aldehyde structure. No detectable MS signals for *m/z* values of C4C6oxG2, C4C6oxG5–6 were found. Overall, MS intensity representing C4C6ox having C6 aldehyde structure was too low to confirm or disapprove route II in Scheme 1.

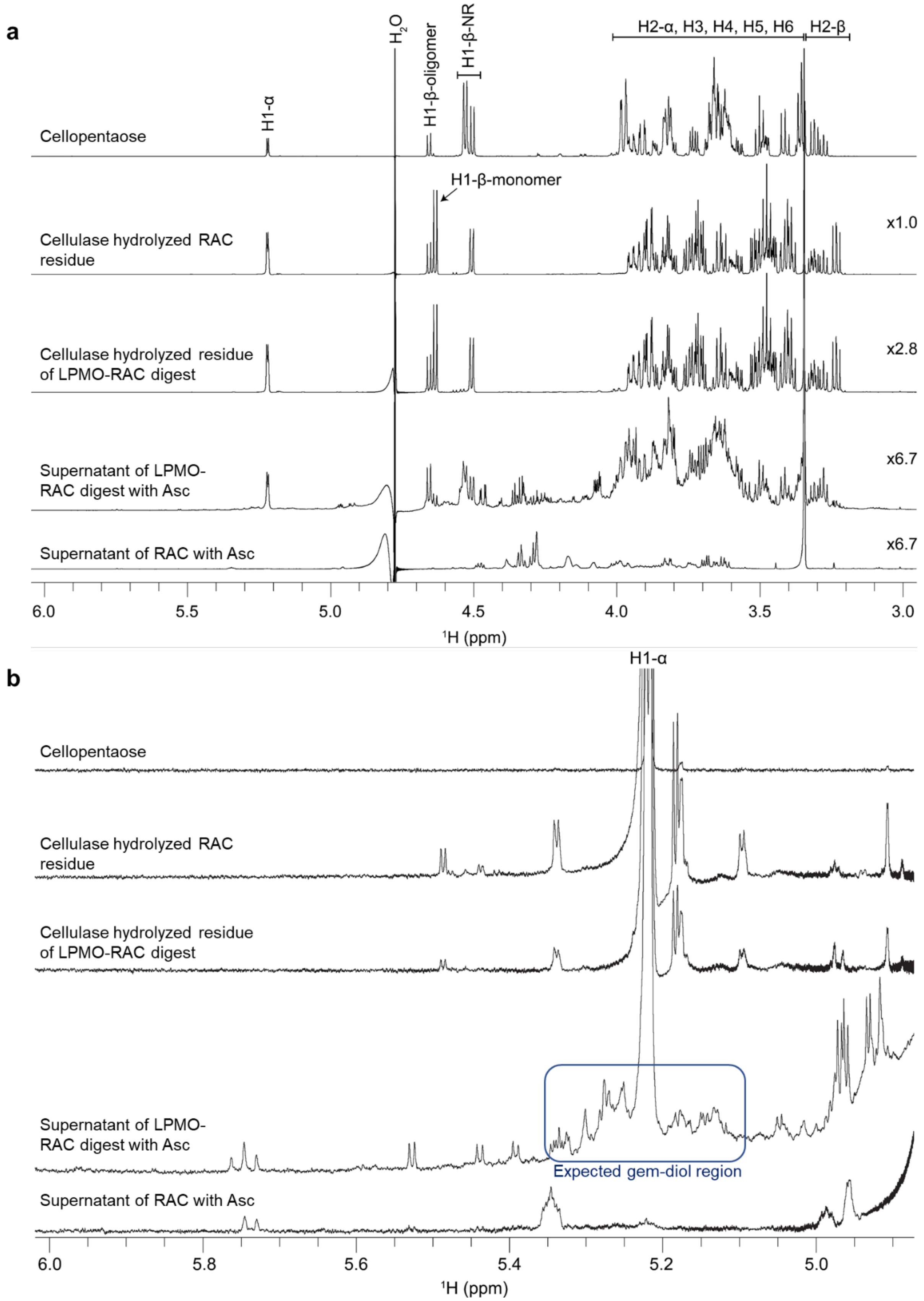

Figure S14. ^1H NMR spectra (700 MHz, D_2O, water suppressed; for experimental setup see Page 3 of this Supporting Information) of LPMO-digested RAC supernatant and residue after cellulase (Celluclast® 1.5L) hydrolysis with (a) 3.0–6.0 ppm region and (b) 4.9–6.0 ppm region of a cellopentaose standard,

residue of untreated RAC hydrolyzed by cellulases, residue of LPMO-RAC digest hydrolyzed by cellulases, LPMO-RAC digest supernatant. Both the control samples for cellulase hydrolysis (cellulase sample only) and for the LPMO-RAC digest supernatant (RAC incubated with ascorbic acid (Asc)) showed no interfering peaks in the regions of interest. The spectra in panel (a) were scaled to get an overall comparable intensity among the spectra, the scaling factor is indicated on the right. In panel (b) no scaling factor was applied.

Annotated chemical shifts are in line with literature.[11] The presence of a C6 gem-diol has previously been shown to result in a clear downfield shift of the H6 proton as compared to non-oxidized glycosyl units, with H6 chemical shifts reported at 5.27 ppm for the gem-diols of methyl-α-D-mannopyranoside and methyl-α-D-glucopyranoside,[12] and 5.11 ppm for the gem-diol of methyl-α-D-galactopyranoside.[13] Given the vast amount of different cello-oligosaccharide structures that can include the C6 gem-diol functional groups in our samples, i.e. gem-diols can be present in combination with either C4 ketones or C4 gem-diols, both at various DP's, we expect a range of chemical shifts rather than a single chemical shift. Evidently, several peaks in the expected gem-diol spectral window (panel b) were present in the LPMO-RAC supernatant, which appeared absent in the reaction control samples and absent in both residues after cellulase hydrolysis. We therefore consider it unlikely that specific C6 oxidation occurs but rather seems coupled to LPMO-cleavage of the β-(1→4)-glucosidic bond.

A signal at 9.36 ppm was detected in the supernatant of the LPMO-RAC digest (not shown), which might correspond to a C6 aldehyde,[13-14] suggesting that C4C6ox having a C6 aldehyde structure might be generated via route II in Scheme 1. However, similar as described in Figure S13, more evidence would be required to prove (or disprove) route II in Scheme 1.

References

[1] M. Frommhagen, S. Sforza, A. H. Westphal, J. Visser, S. W. Hinz, M. J. Koetsier, W. J. H. van Berkel, H. Gruppen, M. A. Kabel, *Biotechnol. Biofuels* **2015**, *8*, 101.

[2] P. Sun, S. V. Valenzuela, P. Chunkrua, F. I. J. Pastor, C. V. F. P. Laurent, R. Ludwig, W. J. H. van Berkel, M. A. Kabel, *ACS Sustain. Chem. Eng.* **2021**, *9*, 14124-14133.

[3] a) P. J. Punt, R. P. Burlingame, C. M. Pynnonen, P. T. Olson, J. Wery, J. Visser, J. Heinrich, M. Emalfarb, J. Visser, J. Verdoes, in *World Intellectual Property Organization patent, Vol. 2*, **2010**; b) H. Visser, V. Joosten, P. J. Punt, A. V. Gusakov, P. T. Olson, R. Joosten, J. Bartels, J. Visser, A. P. Sinitsyn, M. A. Emalfarb, *Ind. Biotechnol.* **2011**, *7*, 214-223.

[4] P. Sun, M. Frommhagen, M. Kleine Haar, G. van Erven, E. J. Bakx, W. J. H. van Berkel, M. A. Kabel, *Carbohydr. Polym.* **2020**, *234*, 115917.

[5] a) R. Kittl, D. Kracher, D. Burgstaller, D. Haltrich, R. Ludwig, *Biotechnol. Biofuels* **2012**, *5*; b) C. V. F. P. Laurent, P. Sun, S. Scheiblbrandner, F. Csarman, P. Cannazza, M. Frommhagen, W. J. H. van Berkel, C. Oostenbrink, M. A. Kabel, R. Ludwig, *Int. J. Mol. Sci.* **2019**, *20*, 6219.

[6] a) M. A. B. Grieco, M. Haon, S. Grisel, A. L. de Oliveira-Carvalho, A. V. Magalhaes, R. B. Zingali, N. Pereira, Jr., J. G. Berrin, *Front. Bioeng. Biotechnol.* **2020**, *8*, 1028; b) C. M. Phillips, W. T. Beeson, J. H. Cate, M. A. Marletta, *ACS Chem. Biol.* **2011**, *6*, 1399-1406.

[7] A. S. Borisova, T. Isaksen, M. Dimarogona, A. A. Kognole, G. Mathiesen, A. Varnai, A. K. Rohr, C. M. Payne, M. Sorlie, M. Sandgren, V. G. H. Eijsink, *J. Biol. Chem.* **2015**, *290*, 22955-22969.

[8] M. Frommhagen, M. J. Koetsier, A. H. Westphal, J. Visser, S. W. Hinz, J.-P. Vincken, W. J. H. van Berkel, M. A. Kabel, H. Gruppen, *Biotechnol. Biofuels* **2016**, *9*, 186.

[9] Z. Forsberg, A. K. Mackenzie, M. Sorlie, A. K. Rohr, R. Helland, A. S. Arvai, G. Vaaje-Kolstad, V. G. H. Eijsink, *Proc. Natl. Acad. Sci. U. S. A.* **2014**, *111*, 8446-8451.

[10] B. Domon, C. E. Costello, *Glycoconjugate J.* **1988**, *5*, 397-409.

[11] T. Koso, D. R. del Cerro, S. Heikkinen, T. Nypelö, J. Buffiere, J. E. Perea-Buceta, A. Potthast, T. Rosenau, H. Heikkinen, H. Maaheimo, *Cellulose* **2020**, *27*, 7929-7953.

[12] T. Breton, G. Bashiardes, J. M. Léger, K. B. Kokoh, *Eur. J. Org. Chem.* **2007**, *2007*, 1567-1570.

[13] K. Parikka, M. Tenkanen, *Carbohydr. Res.* **2009**, *344*, 14-20.

[14] A. C. Boccia, G. Scavia, I. Schizzi, L. Conzatti, *Molecules* **2020**, *25*, 2557.
